# Modeling the Effects of Relapse in the Transmission Dynamics of Malaria Parasites

**DOI:** 10.1155/2012/921715

**Published:** 2011-09-28

**Authors:** Ricardo Águas, Marcelo U. Ferreira, M. Gabriela M. Gomes

**Affiliations:** ^1^Instituto Gulbenkian de Ciência, 2781-901 Oeiras, Portugal; ^2^Department of Parasitology, Institute for Biomedical Sciences, University of São Paulo, 05508-000 São Paulo, SP, Brazil

## Abstract

Often regarded as “benign,” *Plasmodium vivax* infections lay in the shadows of the much more virulent *P. falciparum* infections. However, about 1.98 billion people are at risk of both parasites worldwide, stressing the need to understand the epidemiology of *Plasmodium vivax*, particularly under the scope of decreasing *P. falciparum* prevalence and ecological interactions between both species. Two epidemiological observations put the dynamics of both species into perspective: (1) ACT campaigns have had a greater impact on *P. falciparum* prevalence. (2) Complete clinical immunity is attained at younger ages for *P. vivax*, under similar infection rates. We systematically compared two mathematical models of transmission for both Plasmodium species. Simulations suggest that an ACT therapy combined with a hypnozoite killing drug would eliminate both species. However, *P. vivax* elimination is predicted to be unstable. Differences in age profiles of clinical malaria can be explained solely by *P. vivax*'s ability to relapse, which accelerates the acquisition of clinical immunity and serves as an immunity boosting mechanism. *P. vivax* transmission can subsist in areas of low mosquito abundance and is robust to drug administration initiatives due to relapse, making it an inconvenient and cumbersome, yet less lethal alternative to *P. falciparum*.

## 1. Introduction

*Plasmodium falciparum *has traditionally been the main focus of malaria control programs worldwide, mainly because this parasite is the major cause of severe morbidity and mortality in tropical Africa. However, at a time when global eradication is advocated as the ultimate goal of malaria control strategies worldwide, *P. vivax *needs to be given much more attention from researchers, policy makers, and funding agencies. *P. vivax *is a major public health challenge in Central and South America, the Middle East, Central, South, and Southeast Asia, Oceania, and East Africa, where 2.85 billion people are currently at risk of infection [1] and as many as 250 million infections may be due to this species each year [2]. The emergence of drug-resistant strains and severe (sometimes fatal) disease challenges the traditional view of vivax malaria as a benign infection [3]. 

Although cytoadhesion of *P. vivax-*infected erythrocytes to endothelial cells has been recently demonstrated and might contribute to the pathogenesis of severe vivax malaria [4], this phenomenon is likely to be much less common than with *P. falciparum* infections [5]. Compared to *P. falciparum*, *P. vivax* has a slightly longer incubation period (12 days to several months) and a similarly phased erythrocytic cycle (42–48 hours) that yields fewer merozoites per schizont [6]. However, the distinct ability of *P. vivax* to stay dormant in host's liver cells and cause relapses weeks or months after the primary infection is the most striking difference between *P. vivax* and *P. falciparum*. Although the molecular mechanism of relapse remains undisclosed, progression of the parasite through its life cycle is fairly well described [5, 6]. A proportion of sporozoites remain dormant in the liver, as hypnozoites, for prolonged periods of time before developing and causing recurrent infection. 

The ability to relapse is thought to render *P. vivax* resilient to eradication efforts. In fact, in areas where both species are present, ACT (artemisinin-based combination therapy) campaigns have had a greater impact on *P. falciparum* than on *P. vivax* prevalence [7, 8]. We demonstrate how this is evident when contemplating elimination scenarios, with *P. vivax* elimination being extremely difficult to achieve by mass drug administration. 

Immunity to human malaria is largely species specific. Epidemiological studies have accumulated evidence that clinical (antidisease) and antiparasite immunity is attained at younger ages for *P. vivax*, when compared to *P. falciparum*, under similar infection rates [9–11]. This suggests that immunity is acquired through different mechanisms [12]. We put forward an alternative hypothesis arguing that differences between the observed age profiles lie in the characteristic life cycles of both parasites. Explicitly, we propose that the ability of *P. vivax* to relapse can accelerate the piecemeal acquisition of clinical immunity. 

## 2. Methods

**Figure 1:**
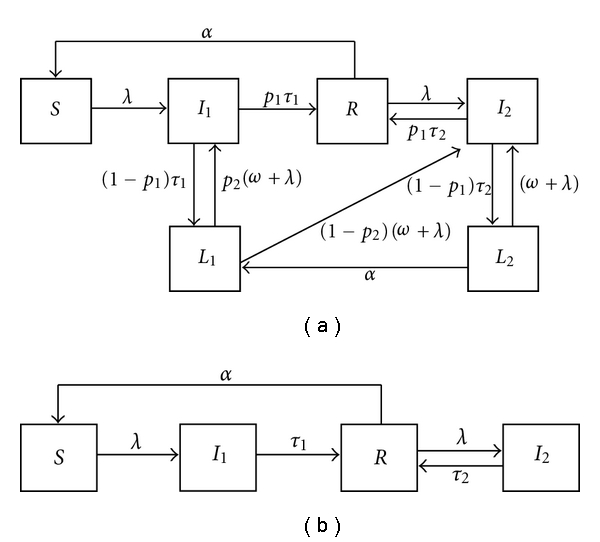
Plasmodium transmission dynamics. (a) Illustration of the natural history of infection with the *P. vivax* parasite. The variables represent a classification of the population at any given age and time into six states: completely susceptible (*S*); clinical malaria resulting from an infection in a completely susceptible individual (*I*_1_); recovered with clinical immunity without any hypnozoites (*R*); mild or asymptomatic infection resulting from exposure of recovered individuals (*I*_2_); recovered with a certain degree of clinical immunity, carrying hypnozoites (*L*_1_); recovered with clinical immunity, carrying hypnozoites (*L*_2_). Description and values for the parameters can be found in Table 1. (b) *P. falciparum* transmission dynamics. This is a subset of the previous system which is retrieved by making *p*_1_ = 1.

We developed a model representing the transmission dynamics of *P. vivax* by adding new elements to the foundation laid by previous work in *P. falciparum* [13]. We have thus a model structure for *P. vivax* transmission, represented by Figure 1(a), which contains the topology representing *P. falciparum* as a submodel (Figure 1(b)). The falciparum dynamics are retrieved by equating *p*_1_ to 1 in the *P. vivax* model. Natural history of *P. vivax* infection is generally similar to that of *P. falciparum*, with a few but crucial idiosyncrasies. 

Susceptible individuals (*S*) are subject to a certain rate of infection (here represented by the force of infection Λ), which depends on local environmental and socioeconomic factors. In *P. vivax*, after a mosquito infectious bite, an indeterminate proportion of the inoculated sporozoites remains dormant in the liver, whilst the remaining develops into erythrocyte invading merozoites. The model describing the transmission dynamics of *P. vivax* must then include a latent class, representing those individuals who, after recovering from infection, keep a remnant of dormant liver forms, called hypnozoites (subject to reactivation at rate *ω*) rather than clearing all parasites while acquiring clinical immunity. Reactivation is still a rather cryptic process, and most relapses seem to result from activation of heterologous hypnozoites [14], which suggests that genotype-specific immunity somehow modulates the occurrence of relapses, much to the resemblance of how the clinical outcome of a given infection is determined [15]. Here, parameter *p*_1_ accounts for episodes not followed by a relapse, either because no hypnozoites were formed or because the remaining hypnozoites do not reactivate during their lifespan. For illustration purposes, throughout the paper we keep *p*_1_ = 0.25 for *P. vivax* (and *p*_1_ = 1 for *P. falciparum*). 

It is generally accepted that the severity of malaria episodes decreases as the host accumulates exposures to the parasite. We implement this aspect of malaria immunity by discretizing the malaria clinical spectrum into two compartments: *I*_1_ represents the severe end of the spectrum, and is labeled “clinical malaria," while *I*_2_ represents the less severe part and is labeled “asymptomatic malaria". Naturally, there is a degree of arbitrariness in this compartmentalization, and the results should be interpreted in this context. We consider that immunologically naïve individuals will display clinical symptoms when infected (*I*_1_). Although *P. vivax* infections are generally not as severe as those caused by *P. falciparum*, they are far from benign. Community studies have revealed that the proportion of *P. vivax* infections presenting with fever is similar to the one registered for *P. falciparum* [16]. Relapse originates either new clinical episodes or asymptomatic infections in accordance with empirical data [17]. The probability of clinical outcome upon relapse in individuals that kept hypnozoites (*L*_1_) from a previous clinical infection is determined by parameter *p*_2_. Latent individuals carrying hypnozoites are subject to reinfection at rate Λ, with the resultant infection phenotype being determined by parameter *p*_2_ as well. 

Individuals who have just recovered from a clinical malaria episode are said to have acquired temporary clinical immunity (*R*). This means that they do not display clinical symptoms upon reinfection and that, unless they are challenged again within a given time frame, they will lose that clinical protection. The same applies to those who have just recovered from an asymptomatic infection. In fact these infections are crucial in boosting acquired clinical immunity, and the interplay between the rate of infection in clinically immune individuals and the rate of clinical immunity loss is fulcrum in determining the number of expected clinical malaria cases during one's lifespan [18]. As such, those recovering from an asymptomatic infection are also subject to loss of immunity at rate *α*. These include individuals that either clear all parasite forms and return to *R* (a proportion *p*_1_) or keep a remnant of hypnozoites and go to the *L*_2_ class. We consider that, in the latter case, subsequent infections and relapse will give rise to asymptomatic malaria. 

The described dynamics can be written as the following system of differential equations:

(1) $$\begin{array}{cc} \frac{\partial S}{\partial t}+\frac{\partial S}{\partial a} & =\alpha R-\left(\lambda \left(a\right)+\mu \right)S,\\ \frac{\partial I_{1}}{\partial t}+\frac{\partial I_{1}}{\partial a} & =\lambda \left(a\right)S+p_{2}\left(\omega +\lambda \left(a\right)\right)L_{1}-\left(\tau_{1}+\mu \right)I_{1},\\ \frac{\partial R}{\partial t}+\frac{\partial R}{\partial a} & =p_{1}\tau_{1}I_{1}+p_{1}\tau_{2}I_{2}-\left(\alpha +\lambda \left(a\right)+\mu \right)R,\\ \frac{\partial I_{2}}{\partial t}+\frac{\partial I_{2}}{\partial a} & =\lambda \left(a\right)R+\left(\omega +\lambda \left(a\right)\right)L_{2}\\ & \;+\left(1-p_{2}\right)\left(\omega +\lambda \left(a\right)\right)L_{2}-\left(\tau_{2}+\mu \right)I_{2},\\ \frac{\partial L_{1}}{\partial t}+\frac{\partial L_{1}}{\partial a} & =\left(1-p_{1}\right)\tau_{1}I_{1}+\alpha L_{2}-\left(\omega +\lambda \left(a\right)+\mu \right)L_{1},\\ \frac{\partial L_{2}}{\partial t}+\frac{\partial L_{2}}{\partial a} & =\left(1-p_{1}\right)\tau_{2}I_{2}-\left(\omega +\lambda \left(a\right)+\alpha +\mu \right)L_{2}, \end{array}$$

with boundary conditions at age *a* = 0: *S*(*t*, 0) = *μ* and *I*_*i*_(*t*, 0) = *L*_*i*_(*t*, 0) = *R*(*t*, 0) = 0 for *i* = 1,2.

The *P. falciparum* transmission dynamics are retrieved by making *p*_1_ = 1:

(2) $$\begin{array}{c} \frac{\partial S}{\partial t}+\frac{\partial S}{\partial a}=\alpha R-\left(\lambda \left(a\right)+\mu \right)S,\\ \frac{\partial I_{1}}{\partial t}+\frac{\partial I_{1}}{\partial a}=\lambda \left(a\right)S-\left(\tau_{1}+\mu \right)I_{1},\\ \frac{\partial R}{\partial t}+\frac{\partial R}{\partial a}=\tau_{1}I_{1}+\tau_{2}I_{2}-\left(\lambda \left(a\right)+\alpha +\mu \right)R,\\ \frac{\partial I_{2}}{\partial t}+\frac{\partial I_{2}}{\partial a}=\lambda \left(a\right)R-\left(\tau_{2}+\mu \right)I_{2}. \end{array}$$

The force of infection was constructed as an age-dependent parameter [13]

(3) $$\lambda \left(a\right)=\lambda_{0}\left(1-ce^{-ka}\right).$$

The function is strictly increasing with age, with a minimum *λ*_0_(1 − *r*) (at age zero) converging asymptotically to *λ*_0_ as age increases. Parameter *k* determines how steeply the force of infection increases with age, and *r* controls the magnitude of that increase. A summary measure of transmission is obtained by integrating the force of infection over age as

(4) Λ=∫λ(a)P(a)da,

where *P*(*a*) = *μe* ^−*μa*^ is the total population distributed over age and *μ* is the birth and death rate. Adopting standard assumptions, Λ is proportional to the frequency of infectious individuals, the proportionality constant being the transmission coefficient,

(5) $$\beta =\frac{\Lambda }{I_{1}+I_{2}}.$$

This standard assumption allows us to analyze how the equilibrium behavior of the system depends on *β*, which is a critical transmission parameter, representing the sylvatic portion of the classical Macdonald formulation of the basic reproduction number for vector-borne diseases [19]. The basic reproduction number for the *P. vivax *model presented here assumes the form

(6) $$\begin{array}{c} R_{0}=\frac{\beta \left(\varepsilon p_{2}N_{1}+\tau_{1}\varepsilon p_{1}N_{2}+\tau_{2}\varepsilon p_{1}N_{3}+\mu \left(\varepsilon +\mu \right)N_{4}+\tau_{1}\varepsilon N_{5}\right)}{\varepsilon^{2}\mu D_{1}+\varepsilon \mu \tau_{2}D_{2}+\tau_{1}\alpha D_{3}+\tau_{1}\varepsilon^{2}p_{1}D_{4}+\mu^{2}D_{5}+\mu^{3}D_{6}}, \end{array}$$

where

(7) $$\begin{array}{cc} N_{1} & =\tau_{1}\left(1-p_{1}\right)\left(-\alpha -\mu \right)+\alpha \tau_{2},\\ N_{2} & =\varepsilon p_{2}-\alpha -\mu ,\\ N_{3} & =\mu +\alpha +\varepsilon -\alpha p_{2},\\ N_{4} & =\left(\varepsilon +\mu \right)+\alpha +\tau_{2},\\ N_{5} & =\alpha +\mu -\varepsilon \left(p_{1}+p_{2}\right)+\varepsilon ,\\ D_{1} & =\tau_{1}+\tau_{2}p_{1}-\tau_{1}p_{2},\\ D_{2} & =\alpha \left(p_{2}+p_{1}-p_{1}p_{2}\right)+\tau_{1}\left(1+p_{1}p_{2}+p_{1}-p_{2}\right),\\ D_{3} & =\mu \left(\varepsilon +\tau_{2}-p_{2}\varepsilon +p_{2}\varepsilon p_{1}\right)+\varepsilon \tau_{2}p_{1},\\ D_{4} & =\tau_{2}-p_{2}\tau_{2}+p_{2}\mu +p_{2}\tau_{2}p_{1},\\ D_{5} & =\left(\tau_{1}+\varepsilon \right)\left(\alpha +\tau_{2}\right)+\tau_{2}\left(\alpha +\varepsilon p_{1}\right)\\ & \;+\varepsilon \tau_{1}\left(2-p_{2}-p_{2}p_{1}+\varepsilon^{2}\right),\\ D_{6} & =\mu +\tau_{2}+\alpha +\tau_{1}+2\varepsilon . \end{array}$$

Epidemiological changes are often attributed to thresholds in transmission, and these are detected through longitudinal trends and comparative studies across multiple communities. We consider the transmission coefficient, *β* (or the basic reproduction number, *R*_0_) as a control parameter and describe the significance of these indices of transmission on selected epidemiological variables. To do so, we calculated the endemic equilibria for systems (1) and (2), without age dependence. Age profiles were obtained by following a cohort under the pressure of an age-dependent force of infection defined by (3), for 20 years. The common variables between systems (1) and (2) have the same boundary conditions. We used the escalator boxcar train (EBT) technique to simulate the dynamics in our age-structured population [20].

To assert the benefits of specific interventions we first simulate our age-structured model in equilibrium conditions to obtain the age profile of clinical disease prevalence without intervention. We use that age profile as the initial condition for the simulation in which the drug administration trial is in vigor. The simulated antimalarial treatment consists of treating every infected individual, regardless of symptoms. In such a setup, asymptomatic malaria is treated as effectively as clinical malaria, and thus the recovery rate from an asymptomatic infection, *τ*_2_, takes the same value as the recovery rate from a clinical infection, *τ*_1_.

## 3. Results

**Figure 2:**
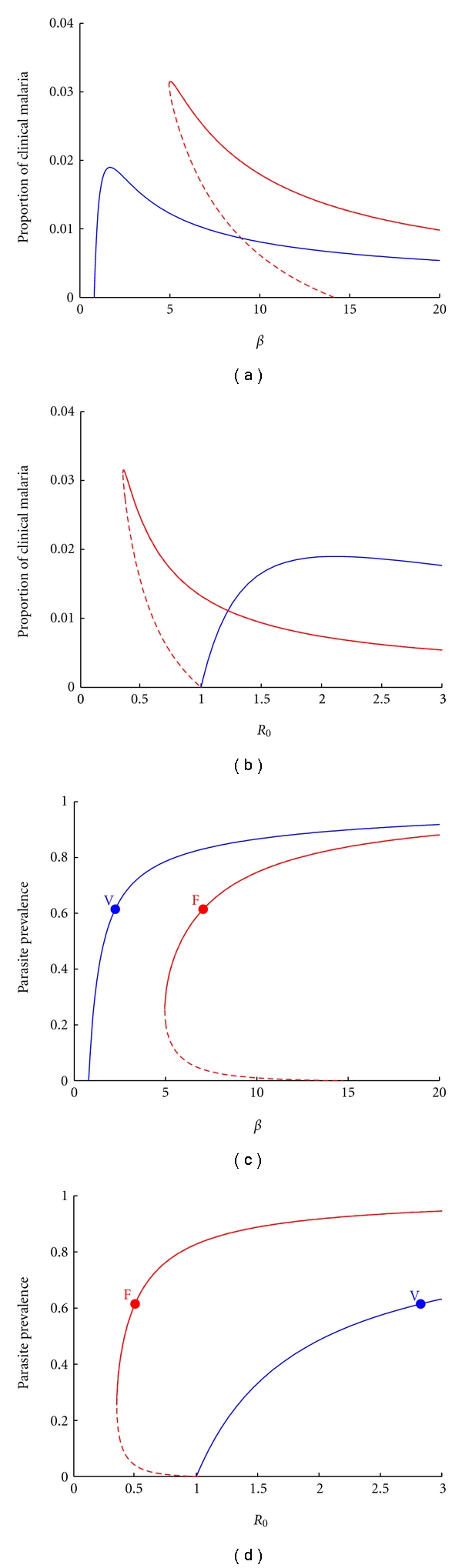
Expected clinical malaria episodes and parasite prevalence at equilibrium, for both *P. vivax* and *P. falciparum*. (a) Bifurcation diagram, showing the influence of *β* on the equilibrium levels of clinical malaria for* P. vivax *(blue) and *P. falciparum* (red). (c) Influence of *β* on the equilibrium levels of parasite prevalence (*I*_1_ + *I*_2_) for* P. vivax *(blue) and *P. falciparum* (red). (b) and (d) are similar to (a) and (c), respectively, but use *R*_0_ as control parameter. For *P. vivax*, we used *p*_1_ = *p*_2_ = 0.25. Dashed lines represent unstable equilibrium solutions, whilst full lines refer to stable endemic equilibria.

The expected prevalence of malaria cases of each Plasmodium species is highly dependent on local environmental and socioeconomic factors that can be summarized into some transmission index. In Figures 2(a) and 2(c), infectious proportions are plotted in terms of a transmission coefficient, *β*, which encapsulates information on contact rates and infectivity. Comparing the model outputs for the *P. falciparum* (red) and *P. vivax* (blue) systems, we verify that, if a significant proportion of individuals recovers from infection with dormant forms of the parasite which reactivate later on, it is easier for the parasite to be transmitted in a sustainable manner (Figure 2(a)). Latency then generates a mechanism by which the parasite population can be maintained in scenarios where vectors are not very abundant or where human-mosquito contacts are sparse. Strikingly, for any value of transmission coefficient that sustains both species, the proportion of individuals with a clinical episode due to *P. vivax* is lower than that due to *P. falciparum* (Figure 2(a)), while the overall parasite prevalence (measured as proportion of individuals in the population which carry parasites in the blood stream and are thus potentially infectious) is expected to be higher for *P. vivax* (Figure 2(c)). Figures 2(b) and 2(d) display how equilibrium solutions depend on the basic reproduction number, *R*_0_, as a proxy for transmission. 

**Figure 3:**
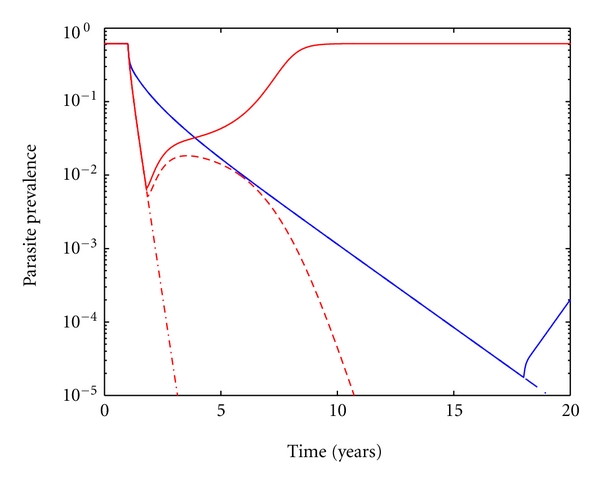
Mass drug administration (MDA) strategy applied in regions where *P. falciparum* (red) and *P. vivax* (blue) are equally prevalent. The initial parasite prevalence is equal for both *P. vivax* and *P. falciparum *as highlighted in Figures 2(c) and 2(d) by V and F, respectively. Asymptomatic infections are treated at a constant rate so they, on average, last as long as a clinical case. The red solid and dashed lines display interventions with durations just below and above the deterministic elimination threshold, respectively, for *P. falciparum*. For *P. vivax*, the model indicates no deterministic elimination threshold. The blue solid line represents continuing the intervention for 18 years and then halting, under *p*_1_ = *p*_2_ = 0.25. The blue and red dot-dashed lines represent uninterrupted 20-year MDA campaigns.

Figure 3 simulates the introduction of malaria control measures in an area supporting *P. vivax* transmission versus in an area supporting *P. falciparum* transmission, with the same parasite prevalence. The intervention consists of applying an MDA (mass drug administration) campaign using ACT aimed at reducing the infectious period of asymptomatic infections, making all infections, regardless of clinical outcome, last the same. Figure 3 portrays that by treating asymptomatic infections equally for both species one can reduce falciparum prevalence (red lines) to a much greater extent than vivax prevalence (blue lines) within the same time frame (dot-dashed lines). This intervention alone leads to sustained elimination of *P. falciparum* if implemented for 274 days (time for the system to enter the basin on attraction of the disease-free equilibrium). In the case of *P. vivax*, elimination is only possible through a stochastic event, and even then the risk of reemergence will be high as the disease-free equilibrium is unstable for this system. If a drug that can eliminate the dormant forms of the parasite (Primaquine) is included in the MDA strategy, then the *P. vivax* dynamics approach those for *P. falciparum*. However, once the intervention is halted the risk for reemergence would still be much higher for *P. vivax* due to instability of the disease-free equilibrium.

**Figure 4:**
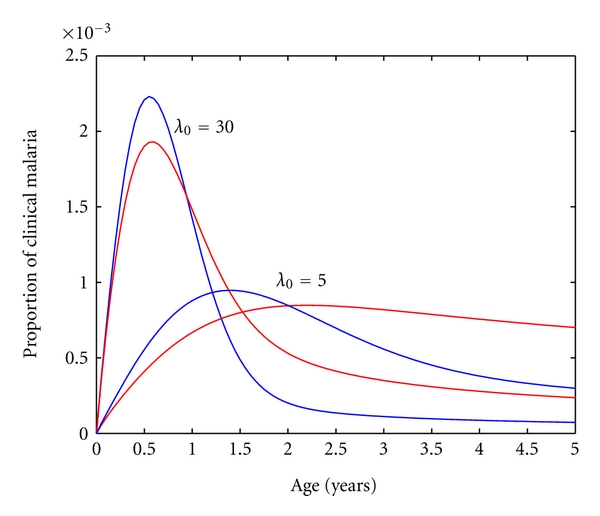
Age profiles for two transmission settings. Clinical *P. vivax* malaria age profiles (blue lines) compared with *P. falciparum* profiles (red lines) for equal risks of infection.

In Figure 4, we simulate the age profiles for *P. vivax* and *P. falciparum* for an equal risk of infection (*λ*_0_). The predicted age profiles for *P. vivax* (blue) display a higher value for clinical malaria cases at the peak, when compared with *P. falciparum* (red), and reveal a decrease in the average age at infection. This means that the risk of a *P. vivax* episode relative to the risk of having a *P. falciparum* episode is greater in very young children and lower throughout childhood. This is consistent in the two transmission settings chosen for this illustration as well as for the entire transmission spectrum.

## 4. Discussion

The true impact of *P. vivax* transmission on human populations stands in the shadow of the overwhelming mortality and morbidity burden exerted by *P. falciparum* worldwide. *P. vivax* importance has been increasingly recognized over the years, and new estimates of the global malaria burden revealed that there are slightly more people at risk of having a *P. vivax* infection than a *P. falciparum* infection [1]. However, probably more interesting is to consider the importance and impact of *P. vivax* under the scope of its ecological interactions with *P. falciparum*, especially considering that coinfection with these species might somehow modulate the clinical outcome of infection [21–23], and that there might be cross-specific immunity [24–27]. Understanding the transmission dynamics of *P. vivax* is crucial to understand the current epidemiological scenario and the potential long-term impact of control interventions. 

We have previously developed a mathematical model to represent the dynamics of *P. falciparum* transmission in human populations [13]. The model was calibrated on hospitalization data from 8 endemic regions in sub-Saharan Africa [28], estimating a fundamental difference between the duration of clinical and asymptomatic infections. This result led to the identification of a deterministic elimination threshold for *P. falciparum* malaria in areas of low to moderate transmission. We adopted the same generic model for *P. vivax* while adding latency classes to represent those individuals who recover from infection with a remnant of dormant parasites, called hypnozoites. These parasites can reactivate at any given time causing a relapse. 

Model outputs were generated and compared with and without latent classes, to mimic vivax and falciparum, respectively, while all other features were unchanged. The expected levels of clinical episodes of both *P. vivax* and *P. falciparum* for the same levels of the transmission coefficient, *β*, indicate that *P. vivax* transmission can be sustained for much lower values of this parameter, when compared with *P. falciparum* (Figure 2(a)). This becomes intuitive in light of *P. vivax*'s ability to relapse, which can transform a single infectious bite into more than one malaria episode leading to higher parasite prevalence in the *P. vivax* system (Figure 2(c))*.* These relationships are inverted, however, when parasite prevalence is represented against the basic reproduction number, *R*_0_, (Figures 2(b) and 2(d)) attesting the importance of standardizing transmission indices. More importantly, we have uncovered a qualitative change due to the latency classes. The deterministic elimination threshold described for the *P. falciparum* system is no longer present under the conditions simulated for *P. vivax*. The parameter regime sustaining the bistability phenomenon that gives rise to the elimination threshold is contracted when latency comes into play.

A major advance in the search for effective malaria drug treatment, following the demise of most known drugs in the battle against resistant parasites, came in the form of artemisinin, a very potent and effective drug against chloroquine and sulphadoxine-pyrimethamine-resistant infections, which can clear parasites and resolve fever faster than any other licensed antimalarial [29]. However, artemisinin derivatives have a very short half-life, translating into substantial treatment failures when used as monotherapy [30], which motivated the combination of artemisinin with longer-lasting partner drugs in the so-called Artemisinin Combination Therapies (ACTs), assuring that there is substantial antimalarial pressure to deal with the residual parasite biomass that may persist when the artemisinin derivatives have fallen below therapeutic levels [31]. Curiously, empirical studies have revealed that the deployment of ACT as a control strategy affects *P. falciparum* transmission much more than it does *P. vivax* transmission [8, 9]. Furthermore, unexpected resurgences of *P. vivax* malaria in areas where elimination attempts were thought to have been successful question to what extent *P. vivax* control is sustainable [32, 33]. Figure 3 supports these results in emphatic fashion. Our simulations suggest that, by treating all infections (equally for both species) with an ACT therapy, one can reduce falciparum prevalence to a much greater extent than vivax prevalence (in the same time scale). This is intrinsically associated with ACT's inability to kill the dormant forms of *P. vivax*, which escape drug action, subsist, and can cause a relapse later on, thus sustaining the parasite pool. In such scenario one should invoke the use of drugs targeting hypnozoites (Primaquine is the only approved and available drug at the moment) as a means of counteracting the parasite's ability to relapse. If everyone was to receive a dose of Primaquine to kill the hypnozoite parasite forms, the dynamics would be similar to that of *P. falciparum*. A remaining difference, however, is that *P. vivax* elimination is predicted to be unstable, meaning that any perturbation in the system (introduction of infectious individuals from neighboring populations for instance) would drive it back to the endemic equilibrium. 

Another epidemiological observation that deserves careful consideration is the differential speed at which complete clinical immunity is attained when comparing *P. vivax* to *P. falciparum* for scenarios of equal risk of infection [9–11]. While others have interpreted this phenomenon as an evidence of there being different mechanisms by which immunity is acquired [12], our results suggest that the difference in age profiles of clinical malaria can be accounted for by the intrinsic transmission dynamics inherent to the natural history of infection of each species. In Figure 4, we can clearly see that the model topologies we used to describe the *P. vivax* and *P. falciparum* transmission dynamics can account for differences in the age profiles of clinical malaria, specifically for low levels of *p*_1_ and *p*_2_. We then propose that differences in age profiles of clinical malaria can be explained solely by *P. vivax*'s ability to relapse, which converts a single infectious mosquito bite into one or more malaria infections, thus accelerating the acquisition of clinical immunity. Relapse also serves as an immunity boosting mechanism that prevents onsets of malaria episodes in older ages. 

On a final note, we recall that all parameters other than those governing hypnozoite formation and relapse were unchanged between the two scenarios. *P. vivax* and *P. falciparum* transmission dynamics might, however, be modulated by a number of biological characteristics such as gametocyte production and antigenic variation. Although our model was not designed to explore these processes, some analogies can be made as detailed in the Supplementary Material available online at doi:10.1155/2012/921715. Two aspects are especially urging. First, it should be acknowledged that *P. vivax* produces gametocytes earlier than *P. falciparum* during a human infection and these gametocytes are shorter lived in the bloodstream [34]. As a consequence, vivax patients are expected to have transmitted more by the time malaria infection is confirmed and treatment is provided. Second, the acquisition of immunity to *P. vivax* relies on expectations regarding strain specificity of natural immunity and antigenic similarity between primary infections and relapses that deserve further attention [35, 36].

## Supplementary Material

The Supplementary Materials provide several sensitivity analyses providing support for the parameters used in the main text and standing as proof of the robustness of the results and conclusions of the paper.

## Figures and Tables

**Table 1:** Model parameters.

Parameter	Description	Value
*μ*	Birth and death rate	0.02 years^−1^
*β*	Transmission coefficient	varying
*λ*	Force of infection	varying
*τ*_1_	Recovery rate from clinical infection	14.12 years^−1^
*τ*_2_	Recovery rate from asymptomatic infections	2.23 years^−1^
*ω*	Relapse rate	12 years^−1^
*α*	Rate of loss of clinical immunity	1.07 years^−1^
*p*_1_	Proportion of vivax infections which recover with hypnozoites	varying
*p*_2_	Proportion of relapses which are clinical	varying
