# Supplementary material for: Modeling the Effects of Relapse in the Transmission Dynamics of Malaria Parasites
Source: J Parasitol Res. 2011 Sep 28;2012:921715. doi: 10.1155/2012/921715 (PMC3182068; doi:10.1155/2012/921715)
Supplement: Supplementary file 1 — The Supplementary Materials provide several sensitivity analyses providing support for the parameters used in the main text and standing as proof of the robustness of the results and conclusions of the paper. [file 921715.f1.doc]

**Supplementary Material**

**Modeling the effects of relapse in the transmission dynamics of malaria parasites**

Ricardo Águas^1^, Marcelo U. Ferreira^2^, M. Gabriela M. Gomes^1^

^1^ Instituto Gulbenkian de Ciência, P-2781-901 Oeiras, Portugal.

^2^ *Department of Parasitology, Institute of Biomedical Sciences, University of São Paulo, Brazil.*

Corresponding author: Ricardo Águas (rjaaguas@gmail.com).

In the main text we have assumed that falciparum and vivax dynamics differ exclusively in what concerns relapse. For illustration purposes we have used a set of parameters describing how relapse affects the *P. vivax* parasite’s life cycle. Here we explore the sensitivity of the results to assumptions in the set of relapse related parameters – *p*_1_, *p*_2_ and *ω* - and further differentiate the transmission dynamics of the two parasite species.

## Sensitivity to relapse parameters

In *P. vivax*, after an infectious mosquito bite, an indeterminate proportion of the inoculated sporozoites remain dormant in the liver, whilst the remaining develop into erythrocyte invading merozoites. Those individuals keeping a pool of dormant liver forms, called hypnozoites are subject to both reinfection and reactivation at rate *ω*. Reactivation is still a rather cryptic process, and most relapses seem to result from activation of heterologous hypnozoites [1], which suggests that genotype‐specific immunity somehow modulates the occurrence of relapses, much to the resemblance of how the clinical outcome of a given infection is determined [2].

We developed a model representing the transmission dynamics of *P. vivax* by adding new elements to the foundation laid by previous work in *P. falciparum* [3]. We have a model structure for *P. vivax* transmission, which contains the topology representing *P. falciparum* as a sub-model, retrieved by equating *p*_1_ to 1. Parameter *p*_1_ accounts for the proportion of episodes not followed by a relapse, either because no hypnozoites were formed or because the remaining hypnozoites do not reactivate during their lifespan. The probability of clinical outcome upon relapse is determined by parameter *p*_2_. For illustration purposes, throughout the paper we keep $p_{1}=p_{2}=0.25$ for *P. vivax*. Here we explore how this assumption affects the results. Figures S1 and S2 show how the equilibrium solutions for the *P. vixax* dynamics (both for clinical malaria and parasite prevalence) depend on the values for *p*_1_ and *p*_2_.


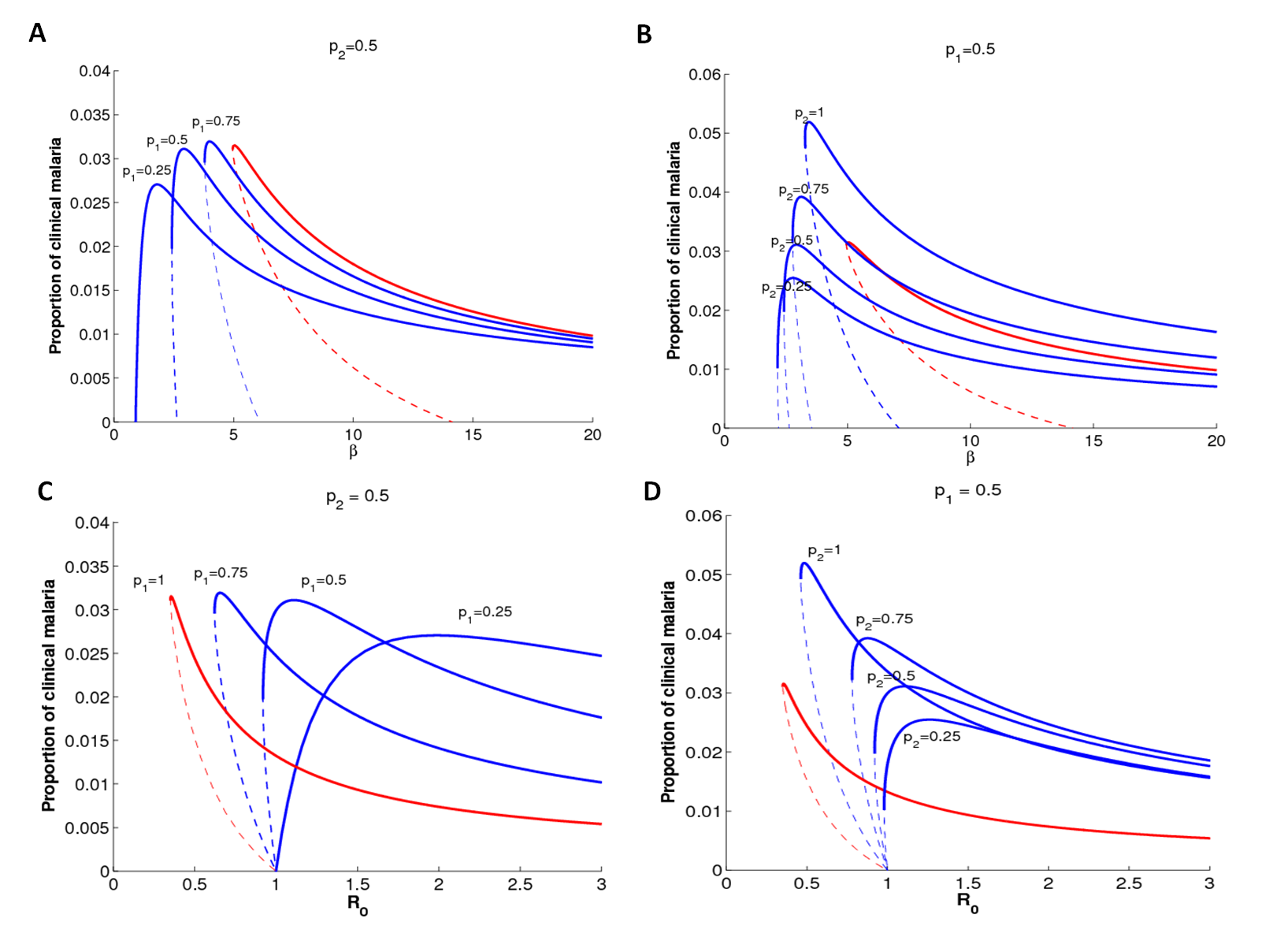


**Figure S1.** **Sensitivity analysis on the expected *P. vivax* clinical malaria episodes at equilibrium.**  **(A, B)** Bifurcation diagrams, showing the influence of *β*, *p*_1_ and *p*_2_ on the equilibrium levels of *P. vivax* clinical malaria. **(C, D)** Bifurcation diagrams, showing the influence of *R*_0_, *p*_1_ and *p*_2_ on the equilibrium levels of *P. vivax* clinical malaria. Throughout, the rate of relapse for *P. vivax*, *ω*, is fixed to be 12 yrs^-1^. The red curves highlight *P. falciparum* equilibria ($p_{1}=1$).


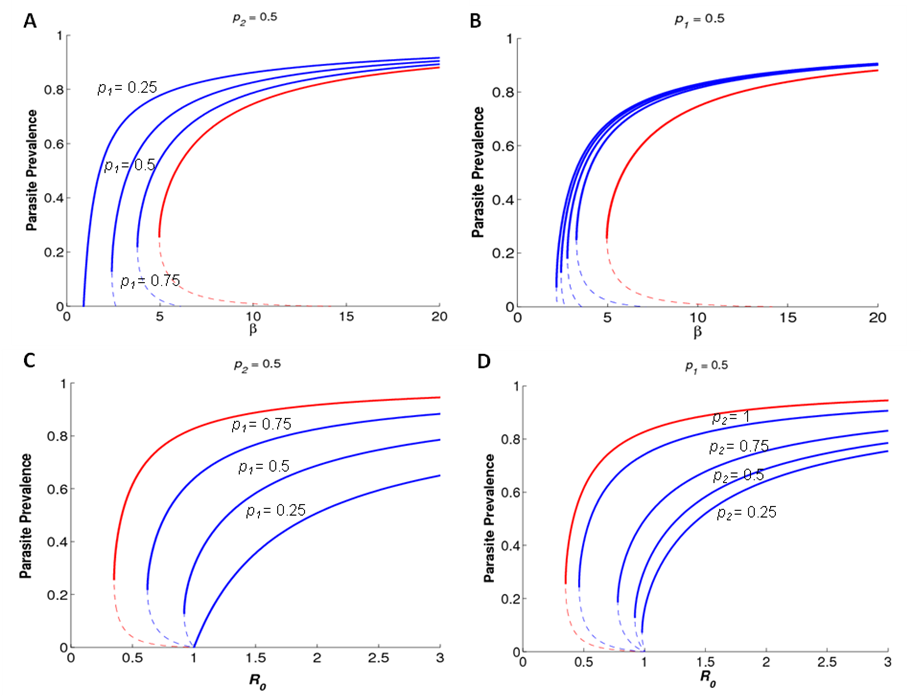


**Figure S2.** **Sensitivity analysis on the expected *P. vivax* parasite prevalence at equilibrium.**  **(A, B)** Bifurcation diagrams, showing the influence of *β*, *p*_1_ and *p*_2_ on the equilibrium levels of *P. vivax* prevalence. In **(B)**, the values of *p*_2_ are increasing from left to right. **(C, D)** Bifurcation diagrams, showing the influence of *R*_0_, *p*_1_ and *p*_2_ on the equilibrium levels of *P. vivax* clinical malaria. Throughout, the rate of relapse for *P. vivax*, *ω*, is fixed to be 12 yrs^-1^. The red curves highlight *P. falciparum* equilibria ($p_{1}=1$).

The actual rate at which relapse occurs appears to depend on geographic and ecological factors. In tropical settings, *P. vivax* relapses are usually 3–6 weeks apart, whereas in temperate areas, there are fewer and more interspersed relapses [4]. Although the relative contributions of reinfection and reactivation are difficult to disentangle, the insensitivity of the model output to changes in the reactivation rate, *ω* (Figure S3) suggests that differences in reinfection rates are likely to be the major determinant for the observed geographical variation in relapse rates.


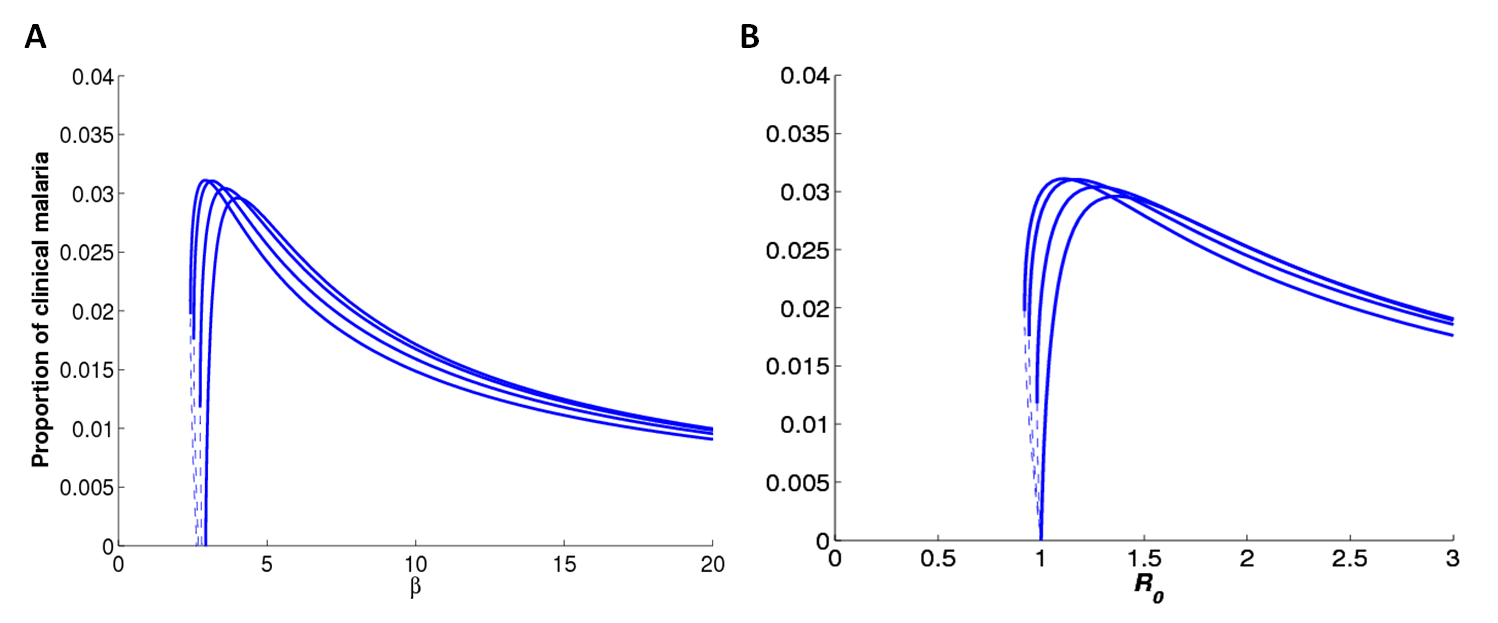


**Figure S3.** **Sensitivity analysis on the expected *P. vivax* clinical malaria episodes at equilibrium.**  **(A)** Bifurcation diagram, showing the influence of *β*, and *ω* on the equilibrium levels of *P. vivax* clinical malaria. Left to right curves correspond to decreasing values (12,8,4,2,1) of the relapse rate, *ω* years^-1^*.* **(B)** Same as in **(A)** while inferring the influence of *R*_0_ on the equilibrium solutions. Throughout,$p_{1}=p_{2}=0.5$

We can visualize the dynamical process by which clinical immunity is acquired through age profiles. In Figure S4 we illustrate how different assumptions regarding relapse probability (*p*_1_) and relapse symptomatology (*p*_2_) shape clinical vivax malaria age profiles. Immunity to *P. vivax* appears to develop at faster rates when compared to *P.* *falciparum* (red curves), given low values for both *p*_1_ and *p*_2_. This reconciles what is known for the *P. vivax* life cycle and observed trends of clinical (anti-disease) and anti-parasite immunity [5-7]. These studies suggest that immunity is attained at younger ages for *P. vivax*, when compared to *P. falciparum*, under similar infection rates. Although this might suggest that different mechanisms of acquisition of immunity are operating, we argue that this phenomenon can be merely a consequence of the different dynamics of transmission for each species stemming from the occurrence of relapses in *P. vivax*.

**Figure S4. Sensitivity analysis on the importance of latency and clinical relapses on *P. vivax* age profiles.** The red line corresponds to the *P. falciparum* age profile. While varying one of the parameters, *p*_1_ or *p*_2_, the other is fixed at 0.25.

Relapse accelerates the rate of acquisition of clinical immunity by re-exposing individuals who have had a previous episode of malaria to new parasites at a frequency which is higher than that expected from the number of infectious mosquito bites received. In some sense, one infectious bite can ultimately be responsible for more than one bout of *P. vivax* parasitaemia, thus boosting clinical immunity more effectively than one infectious bite with *P. falciparum* does. Hence, the more *P. vivax* infections relapse (lower value of *p*_1_) the more pronounced this effect will be, and the more rapidly immunity will be acquired. Parameter *p*_2_ determines the proportion of relapses that give rise to a clinical episode. The effect of this parameter is explored in Figure S4B.

## Sensitivity to rates of loss of infection and immunity

Throughout the paper we have assumed that *P. vivax* and *P. falciparum* transmission dynamics differed exclusively in what concerns relapse. They might, however, differ in a number of biological characteristics such as gametocyte production and antigenic variation. Although our model was not designed to explore these processes, some analogies can be made and assessed to some extent.

Plasmodium vivax produces gametocytes earlier that P. falciparum during a human infection [8]. Moreover, these gametocytes when compared with *P. falciparum* are short-lived in the bloodstream. As a consequence, most vivax malaria patients have circulating gametocytes (therefore being putatively infectious) at the time malaria infection is confirmed and treatment is provided. As a result, treatment of falciparum malaria is often started before gametocytes are produced making the infectious period very short or even non existing. To account for the absence of this early warning in *P. vivax* we now explore the consequences of a slower recovery rate (lower *τ*_1_). As can be seen in Figure S5B there are no significant qualitative effects on the endemic equilibrium curves because the main contributors to transmission under sustained conditions are the asymptomatic infections who do not normally seek treatment. This factor may, however, gain importance under elimination scenarios where treatment rates are likely to increase, as a result of increased surveillance and increased symptomatic fractions due to loss of immunity.


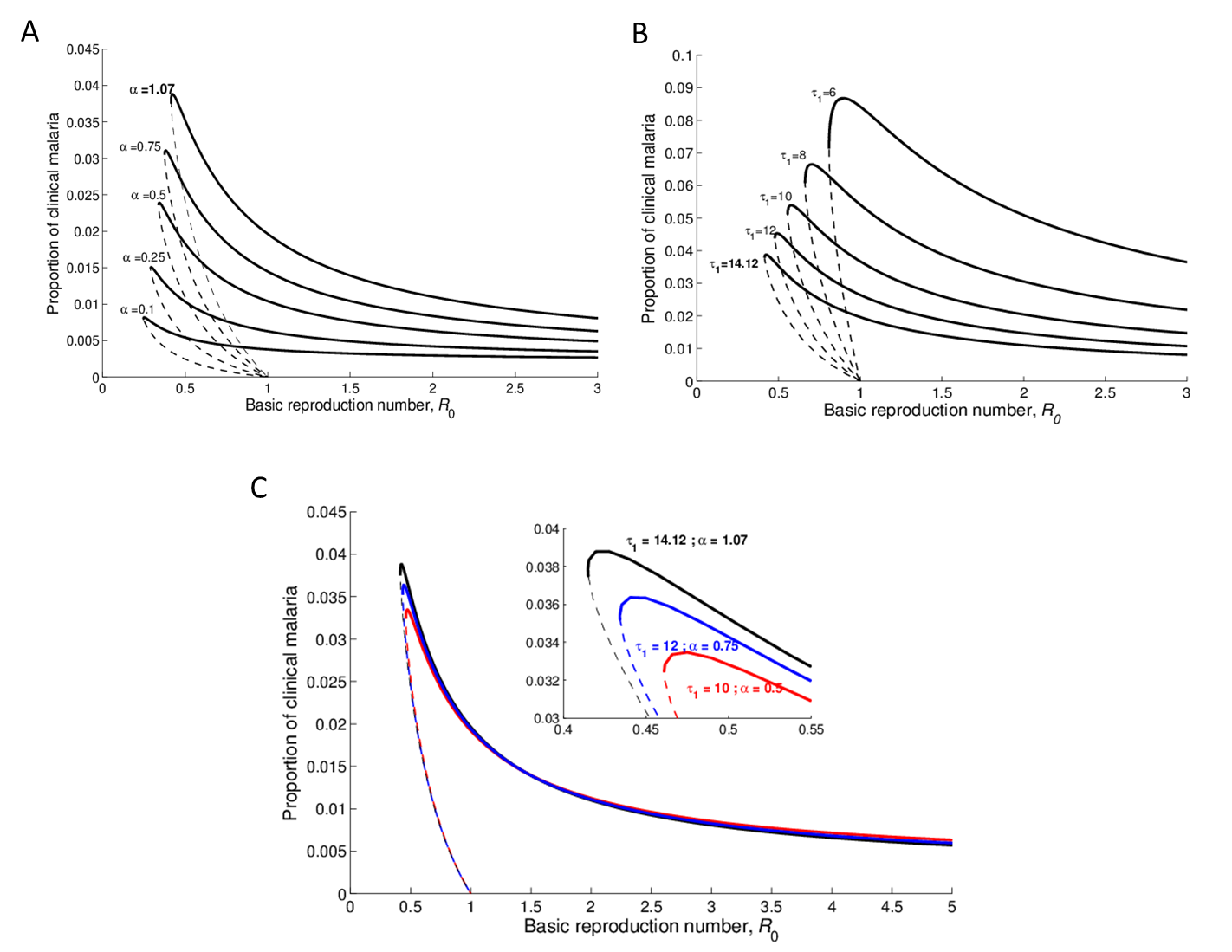


**Figure S5. Effects of the lowering the rates of loss of clinical immunity (*α*) and recovery from infection (*τ*_1_) on the expected proportion of *P. vivax* clinical malaria episodes at equilibrium. (A)** Bifurcation diagram, showing the influence of *α* on the equilibrium levels of *P. vivax* clinical malaria. **(B)** Sensitivity analysis on the influence of *τ*_1_ to the endemic equilibrium solutions. **(C)** Effect of lowering both rates of loss of clinical immunity (*α*) and recovery from infection (*τ*_1_) on the bifurcation plot. The black line corresponds to the values used in the main text.

Classical studies have shown that repeated challenges with the same *P. vivax* strain failed to induce fever in neurosyphilis patients, while infections with the same *P. falciparum* strain were less likely to induce clinical immunity after a few challenges [9]. Since in both examples parasites were genetically identical, antigenic variation (more effective for immune evasion in *P. falciparum*) has been alluded as an explanation the differences observed. The model investigated here does not have control over the number of infections required to induce clinical immunity. To some extent, however, this can be compensated by varying the rate of loss of immunity.

Motivated by these observations, we explore the effects of reducing the rate of loss of immunity (*α*), representing a scenario where more time would be required for immunity to be evaded by any antigenic variant in the case of *P. vivax*. Figure S5A shows the influence of this parameter on the expected proportion of clinical vivax malaria cases. Again, qualitative results are maintained. Naturally, the more effective clinical immunity is, the less clinical cases there will be. Combining the two previous assumptions (differential gametocyte production and antigenic variation), we regenerate the results when values for *τ*_1_ and *α* are lower for *P. vivax* relative to *P. falciparum* (Figure S5C).

One critical point to the validity of our results stands on measured differences in the transmission rates for vivax and falciparum in Michon et al 2007 [5] study area. Michon et al report that risk of re-infection following treatment was roughly 5/child/year for both species using LDR-FMA or 3 for falciparum and 2 for vivax using microscopy. The EIR for falciparum was 37 and 24 for vivax. Motivated by these observations, we explore different scenarios where *P. vivax* transmission is either equal or lower compared to *P. falciparum* transmission (Figure S6). In any case, the less effective acquisition of immunity in the case of *P. falciparum* results from the absence of parasite reactivation.


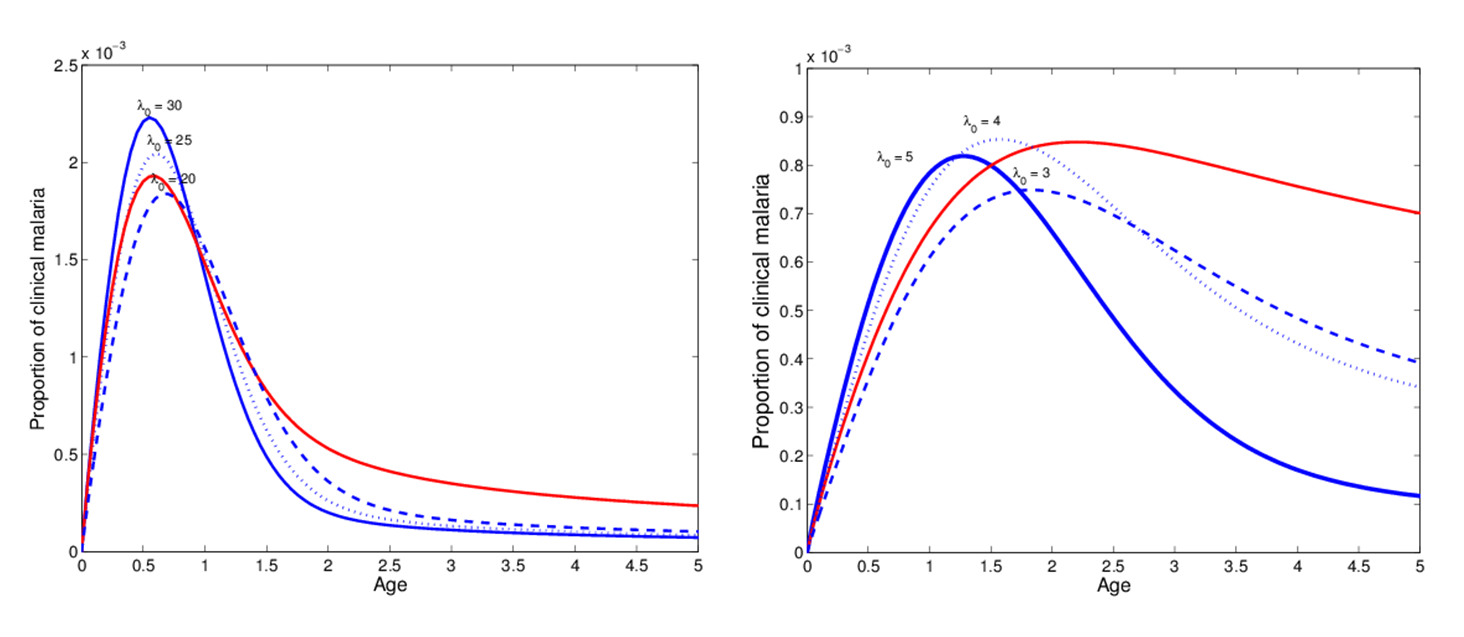


**Figure S6. Exploring differences between *vivax* and malaria age profiles under different transmissions. (A)** The red line corresponds to the *P. falciparum* age profile with$\lambda_{0}=30$. While varying the transmission parameter for *P. vivax* we keep$p_{1}=p_{2}=0.25$. (B) Same as (A) but where the red line corresponds to the *P. falciparum* age profile with$\lambda_{0}=5$.

## References

1. Imwong M, Snounou G, Pukrittayakamee S, Tanomsing N, Kim JR, et al. Relapses of Plasmodium vivax infection usually result from activation of heterologous hypnozoites. J Infect Dis. 2007 Apr 1;195(7):927-33.
2. Osier FH, Fegan G, Polley SD, Murungi L, Verra F, et al. Breadth and magnitude of antibody responses to multiple Plasmodium falciparum merozoite antigens are associated with protection from clinical malaria. Infect Immun. 2008 May;76(5):2240-8.
3. Aguas R, White LJ, Snow RW, Gomes MG. Prospects for malaria eradication in sub-Saharan Africa. PLoS One 2008; **3**(3):e1767.
4. Baird, J.K. et al. Prevention and treatment of vivax malaria. Curr. Infect. Dis. Rep. 2007; 9: 39–46
5. Michon P, Cole-Tobian JL, Dabod E, Schoepflin S, Igu J, Susapu M, Tarongka N, Zimmerman PA, Reeder JC, Beeson JG, Schofield L, King CL, Mueller I. The risk of malarial infections and disease in Papua New Guinean children. Am J Trop Med Hyg. 2007; **76**(6):997-1008.
6. Mendis K, Sina BJ, Marchesini P, Carter R. The neglected burden of Plasmodium vivax malaria. Am J Trop Med Hyg. 2001; **64**(1-2 Suppl):97-106.
7. Genton B, al-Yaman F, Beck HP, Hii J, Mellor S, et al. The epidemiology of malaria in the Wosera area, East Sepik Province, Papua New Guinea, in preparation for vaccine trials. I. Malariometric indices and immunity. Ann Trop Med Parasitol 1995; **89**:359–376.
8. Bousema T and Drakeley C. Epidemiology and Infectivity of Plasmodium falciparum and Plasmodium vivax Gametocytes in Relation to Malaria Control and Elimination. Clinical Microbiology Reviews, 2011,p. 377-410, Vol. 24, No. 2.
9. McKenzie FE, Smith DL, O'Meara WP, Riley EM. Strain theory of malaria: the first 50 years. Adv Parasitol. 2008;66:1-46.
